# Supplementary material for: SLC7A11 expression level dictates differential responses to oxidative stress in cancer cells
Source: Nat Commun. 2023 Jun 21;14:3673. doi: 10.1038/s41467-023-39401-9 (PMC10281978; doi:10.1038/s41467-023-39401-9)
Supplement: Supplementary file 1 — Supplementary Information [file 41467_2023_39401_MOESM1_ESM.pdf]

Supplementary Information

SLC7A11 expression level dictates differential responses to oxidative stress in cancer cells

Yuelong Yan, Hongqi Teng, Qinglei Hang, Lavanya Kondiparthi, Guang Lei, Amber Horbath, Xiaoguang Liu, Chao Mao, Shiqi Wu, Li Zhuang, M. James You, Masha V. Poyurovsky, Li Ma, Kellen Olszewski, Boyi Gan

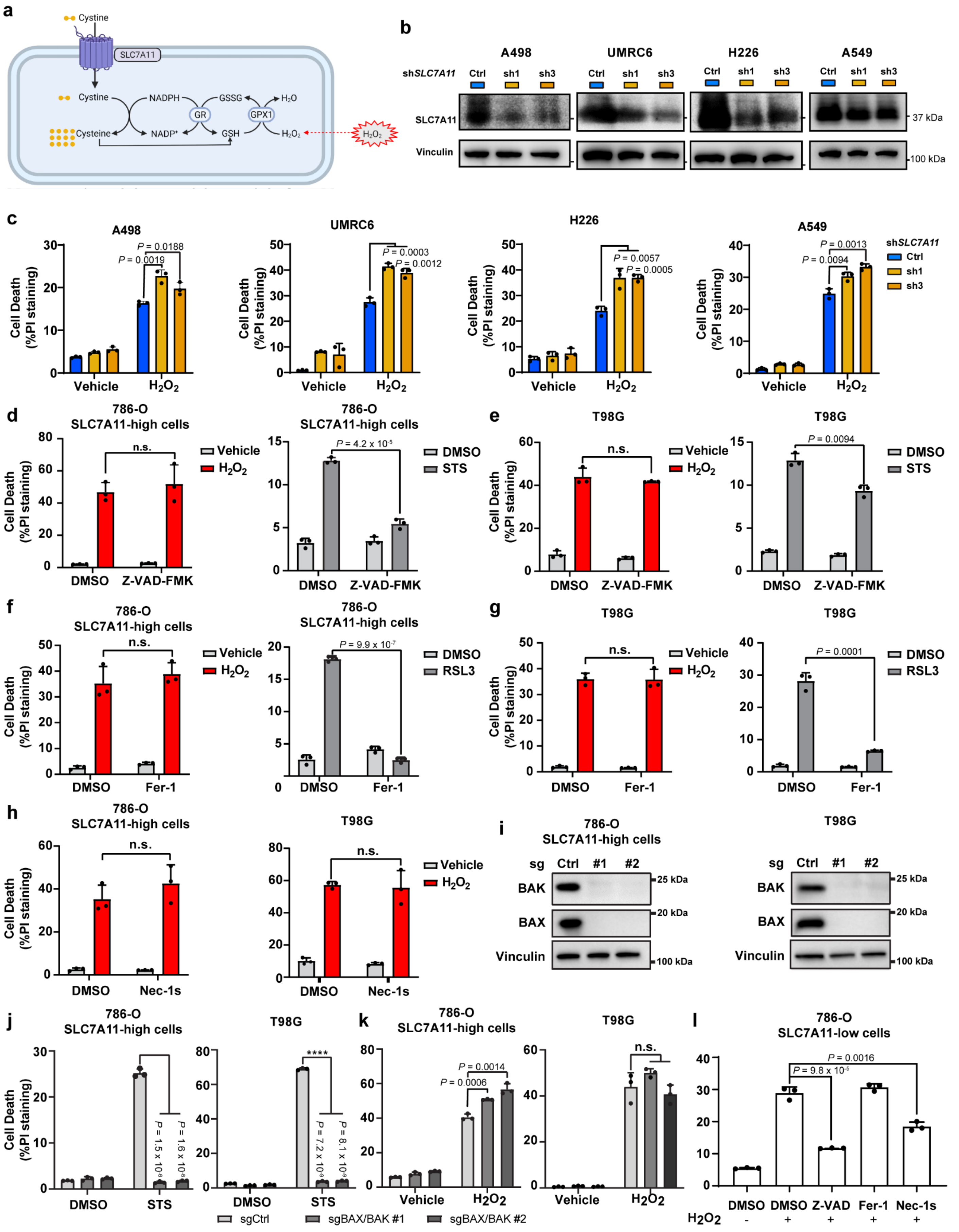
a
Cystine
SLC7A11
Cystine
NADPH
GSSG
H2O
GR
GPX1
Cysteine
NADP+
GSH
H2O2
H2O2
b
A498
UMRC6
H226
A549
shSLC7A11
Ctrl
sh1
sh3
SLC7A11
Vinculin
37 kDa
100 kDa
c
Cell Death (%PI staining)
Vehicle
H2O2
P = 0.0188
P = 0.0019
P = 0.0003
P = 0.0012
P = 0.0057
P = 0.0005
P = 0.0013
P = 0.0094
d
786-O SLC7A11-high cells
n.s.
P = 4.2 x 10-5
DMSO
Z-VAD-FMK
STS
e
T98G
P = 0.0094
f
P = 9.9 x 10-7
Fer-1
RSL3
g
P = 0.0001
h
Nec-1s
i
sg
Ctrl
#1
#2
BAK
BAX
25 kDa
20 kDa
j
P = 1.5 x 10-6
P = 1.6 x 10-6
****
P = 7.2 x 10-9
P = 8.1 x 10-9
sgCtrl
sgBAX/BAK #1
sgBAX/BAK #2
k
P = 0.0014
P = 0.0006
l
786-O SLC7A11-low cells
P = 0.0016
P = 9.8 x 10-5
Z-VAD
H2O2

Supplementary Figure 1. Cell death induced by high SLC7A11 expression under H_2O_2 treatment is not apoptosis, necroptosis, or ferroptosis. a, Simplified schematic showing how cells detoxify H_2O_2 through the SLC7A11-glutathione (GSH)-glutathione peroxidase 1 (GPX1) axis. **b**, Protein levels of SLC7A11 in indicated cell lines with shCtrl and *SLC7A11* knockdown. **c,** Cell death in response to 1 mM H_2O_2 treatment in A498, UMRC6, H226 and A549 cells with indicated genotypes for 24 hours was measured using PI staining. **d-e**, Cell death in response to co-treatment with 1 mM H_2O_2 for 6 hours (left), or 30 μM staurosporine (STS) for 24 hours (right), and 10 μM Z-VAD-FMK in SLC7A11-high 786-O cells **(d)** and T98G cells **(e)** was measured using PI staining. **f**. Cell death in response to co-treatment with 1 mM H_2O_2 for 6 hours (left) or 0.2 μM RSL3 for 16 hours (right) with 5 μM ferrostatin-1 (Fer-1) in SLC7A11-high 786-O cells was measured using PI staining. **g**, Cell death in response to co-treatment with 1 mM H_2O_2 (left) or 1 μM RSL3 (right) for 24 hours with 5 μM ferrostatin-1 in T98G cells was measured using PI staining. **h**, Cell death under co-treatment with 1 mM H_2O_2 and 2 μM necrostatin-1s (Nec-1s) in SLC7A11-high 786-O (left) or T98G (right) cells was measured using PI staining. **i**, Protein levels of BAK and BAX in sgCtrl and *BAX/BAK* double knockout SLC7A11-high 786-O (left) or T98G (right) cells. **j-k**, Cell death in response to treatment with 30 μM STS **(j)** or 1 mM H_2O_2 **(k)** in SLC7A11-high 786-O (left) or T98G (right) cells with indicated genotypes was measured using PI staining. **l,** Cell death under co-treatment with 1 mM H_2O_2 and Z-VAD-FMK, Fer-1 or Nec-1s for 20 hrs. Data were presented as mean ± SD; n = 3. n indicates independent repeats. P value was determined by two-tailed unpaired Student's t test. n.s., not significant. Source data are provided as a Source Data file. GR, glutathione reductase; GSSG, glutathione disulfide; NADPH, nicotinamide adenine dinucleotide phosphate.

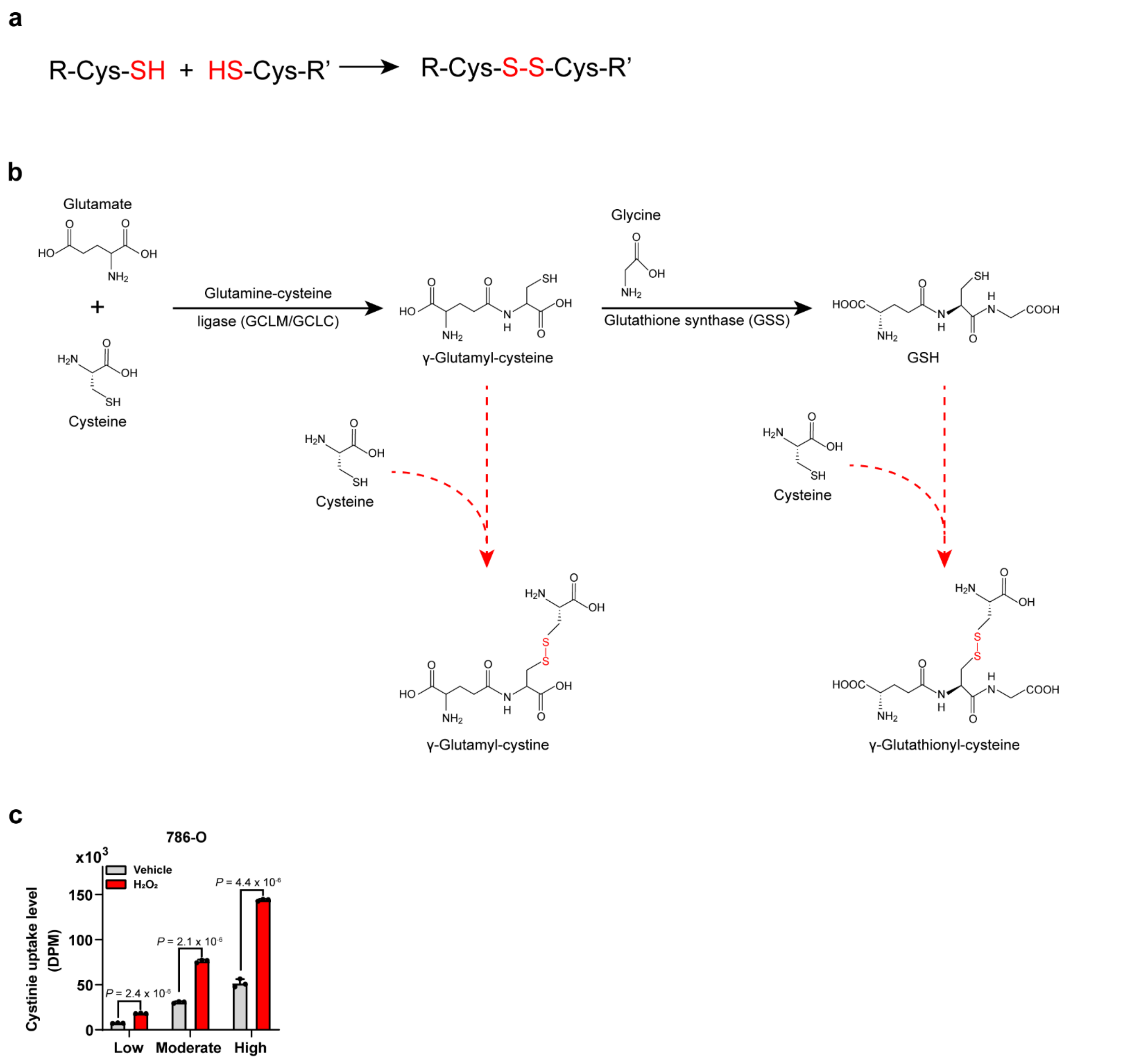

Supplementary Figure 2. Schematic of disulfide molecule formation. a, Diagram illustrating disulfide bond formation. **b**, Diagram illustrating the synthesis of various cystine-derived disulfide molecules. **c,** Cystine uptake levels in SLC7A11-low, -moderate, and -high 786-O cells treated with vehicle or 1 mM H_2O_2 for 1hr. Data were presented as mean ± SD; n = 3. n indicates independent repeats. P value was determined by two-tailed unpaired Student’s t test. Source data are provided as a Source Data file.

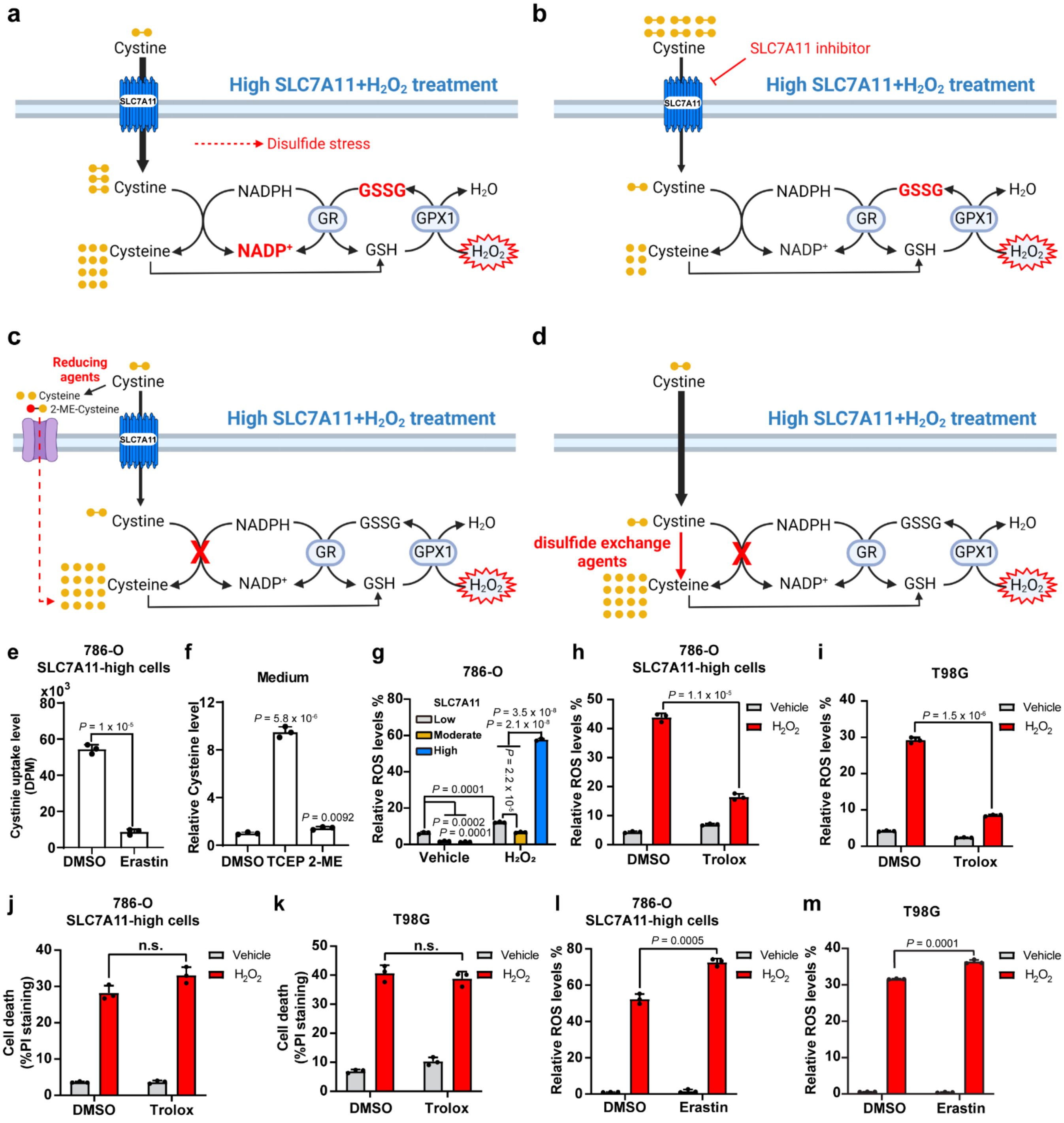

a
Cystine
High SLC7A11+H2O2 treatment
SLC7A11
Disulfide stress
Cystine
NADPH
GSSG
H2O
GR
GPX1
Cysteine
NADP+
GSH
H2O2
b
Cystine
SLC7A11 inhibitor
High SLC7A11+H2O2 treatment
SLC7A11
Cystine
NADPH
GSSG
H2O
GR
GPX1
Cysteine
NADP+
GSH
H2O2
c
Reducing agents
Cystine
Cysteine
2-ME-Cysteine
High SLC7A11+H2O2 treatment
SLC7A11
Cystine
NADPH
GSSG
H2O
GR
GPX1
Cysteine
NADP+
GSH
H2O2
d
Cystine
High SLC7A11+H2O2 treatment
Cystine
disulfide exchange agents
NADPH
GSSG
H2O
GR
GPX1
Cysteine
NADP+
GSH
H2O2
e
786-O SLC7A11-high cells
Cystine uptake level (DPM)
x10^3
P = 1 x 10^-5
DMSO
Erastin
f
Medium
Relative Cysteine level
P = 5.8 x 10^-6
P = 0.0092
DMSO
TCEP
2-ME
g
786-O
Relative ROS levels %
SLC7A11
Low
Moderate
High
P = 3.5 x 10^-8
P = 2.1 x 10^-8
P = 2.2 x 10^-5
P = 0.0001
P = 0.0002
P = 0.0001
Vehicle
H2O2
h
786-O SLC7A11-high cells
Relative ROS levels %
P = 1.1 x 10^-5
Vehicle
H2O2
DMSO
Trolox
i
T98G
Relative ROS levels %
P = 1.5 x 10^-6
Vehicle
H2O2
DMSO
Trolox
j
786-O SLC7A11-high cells
Cell death (%PI staining)
n.s.
Vehicle
H2O2
DMSO
Trolox
k
T98G
Cell death (%PI staining)
n.s.
Vehicle
H2O2
DMSO
Trolox
l
786-O SLC7A11-high cells
Relative ROS levels %
P = 0.0005
Vehicle
H2O2
DMSO
Erastin
m
T98G
Relative ROS levels %
P = 0.0001
Vehicle
H2O2
DMSO
Erastin

Supplementary Figure 3. Uncoupling between reactive oxygen species (ROS) and cell death induced by high SLC7A11 expression under H_2O_2 treatment. a-d, Simplified schematics illustrating redox system alterations in SLC7A11-high cells under H_2O_2 treatment with no additional treatment (**a**), or treatment with SLC7A11 inhibitors (**b**), reducing agents (**c**), or disulfide-exchange agents (**d**). **e**, Cystine uptake level in SLC7A11-high 786-O cells treated with or without 5 µM erastin. **f**, Cysteine level in medium treated with or without 1 mM TCEP or 2-ME. **g**, Relative ROS levels in SLC7A11-low, -moderate, and -high 786-O cells treated with vehicle or 1 mM H_2O_2. **h, i,** Relative ROS levels in SLC7A11-high 786-O (**h**) or T98G (**i**) cells treated with vehicle or 1 mM H_2O_2 with or without 0.5 mM Trolox. **j, k,** Cell death in response to treatment with 1 mM H_2O_2 with or without 0.5 mm Trolox in SLC7A11-high 786-O (**j**) or T98G (**k**) cells measured using PI staining. **l**, **m**, Relative ROS levels in SLC7A11-high 786-O cells (**l**) or T98G (**m**) cells treated with vehicle or 1 mM H_2O_2 (1 mM) with or without 5 µM erastin. Data were presented as mean ± SD; n = 3. n indicates independent repeats. P value was determined by two-tailed unpaired Student's t test. n.s., not significant. Source data are provided as a Source Data file. GPX1, glutathione peroxidase 1; GR, glutathione reductase; GSH, glutathione; GSSG, glutathione disulfide; NADPH, nicotinamide adenine dinucleotide phosphate.

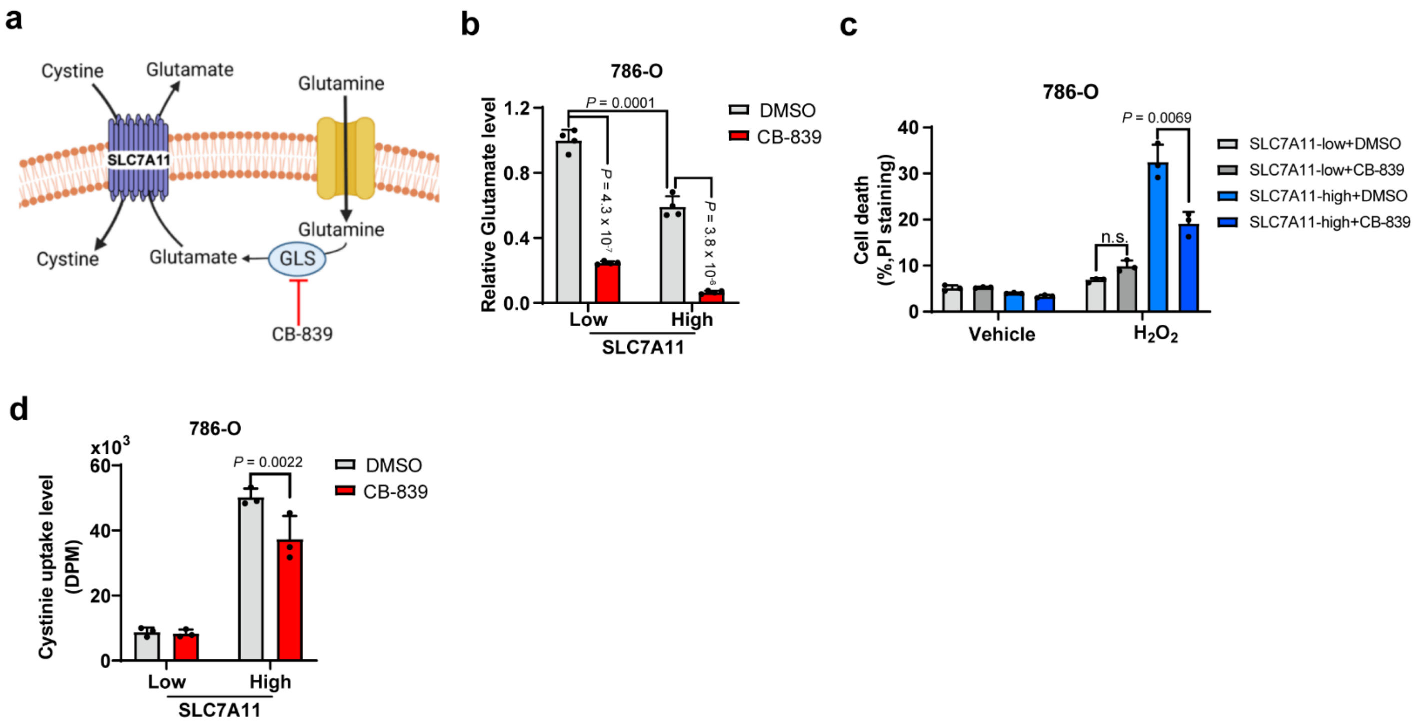

Supplementary Figure 4. Glutaminase inhibition decreases intracellular glutamate levels and suppresses H_2O_2–induced cell death in SLC7A11-high cells. a, Simplified schematic illustrating how glutamine metabolism is involved in SLC7A11-mediated cystine uptake. **b,** Measurement of intracellular glutamate level in SLC7A11-low and -high 786-O cells treated with 5 µM CB-839 for 1 hr (n = 4). **c**, Cell death in response to co-treatment with 1 mM H_2O_2 and 5 µM CB-839 for 6 hr in in SLC7A11-low and -high 786-O cells (n = 3)**. d,** Cystine uptake levels in SLC7A11-low and -high 786-O cells treated with 5 µM CB-839 for 1 hr (n = 3). Data were presented as mean ± SD. n indicates independent repeats. P value was determined by two-tailed unpaired Student's t test. Source data are provided as a Source Data file.

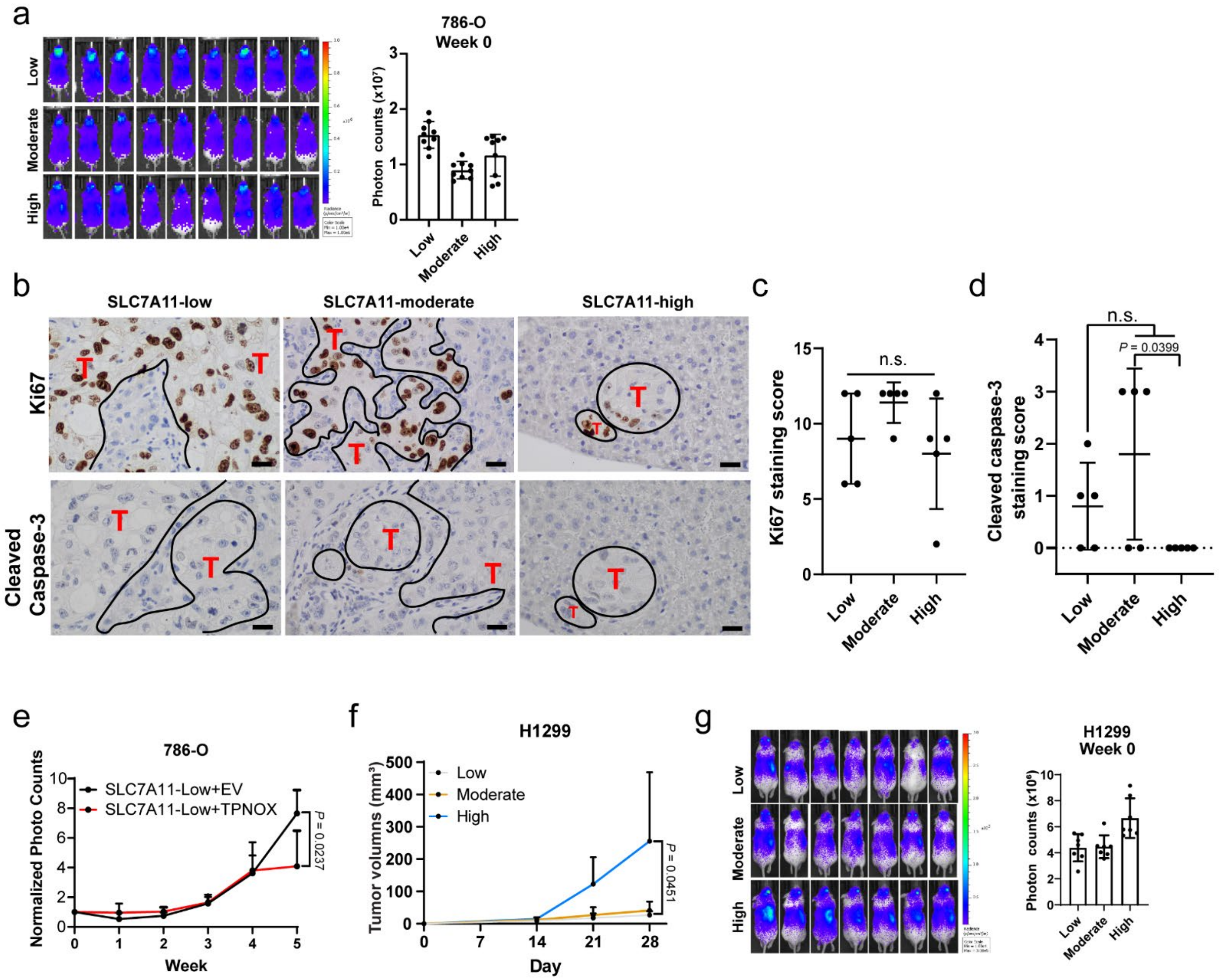
a
Low
Moderate
High
786-O
Week 0
Photon counts (x10⁷)
b
SLC7A11-low
SLC7A11-moderate
SLC7A11-high
Ki67
Cleaved Caspase-3
T
c
Ki67 staining score
n.s.
d
Cleaved caspase-3 staining score
n.s.
P = 0.0399
e
786-O
Normalized Photo Counts
SLC7A11-Low+EV
SLC7A11-Low+TPNOX
P = 0.0237
Week
f
H1299
Tumor volumns (mm³)
Low
Moderate
High
P = 0.0451
Day
g
H1299
Week 0
Photon counts (x10⁶)

Supplementary Figure 5. ***In vivo*** **imaging of cancer cells after intracardiac injection. a,** Images of bioluminescence in mice 30 min after intracardiac injection with SLC7A11-low, -moderate, and -high 786-O cells (left) and statistical analysis of whole-body photon flux (right) (n = 9 mice). **b**, Representative images of immunohistochemical staining (Ki67 and cleaved caspase-3) of livers with tumor metastasis derived from SLC7A11-low, -moderate, or -high 786-O cells. "T" stands for tumor cells. Scale bars, 20 μm. **c, d,** Immunohistochemical staining scores for Ki67 (**c**) and cleaved caspase-3 (**d**) in livers with tumor metastasis derived from SLC7A11-low, -moderate, or -high 786-O cells. **e,** Quantification of photon flux (photons per second) in mice normalized to day 0 after intracardiac injection of SLC7A11-high 786-O cells with EV or TPNOX overexpression (n = 5 mice). **f,** Measurement of tumor volumes in SLC7A11-low, -moderate, and -high H1299 xenograft tumors at different time points (days) after subcutaneous injection (n = 5 mice for low/moderate and n = 4 mice for high). **g,** Images of bioluminescence in mice 30 min after intracardiac injection with SLC7A11-low, -moderate, and -high H1299 cells (left) and statistical analysis of whole-body photon flux (right) (n = 7 mice). Data were presented as mean ± SD. n indicates mice or independent repeats. P value was determined by two-tailed unpaired Student's t test. n.s., not significant. Source data are provided as a Source Data file.

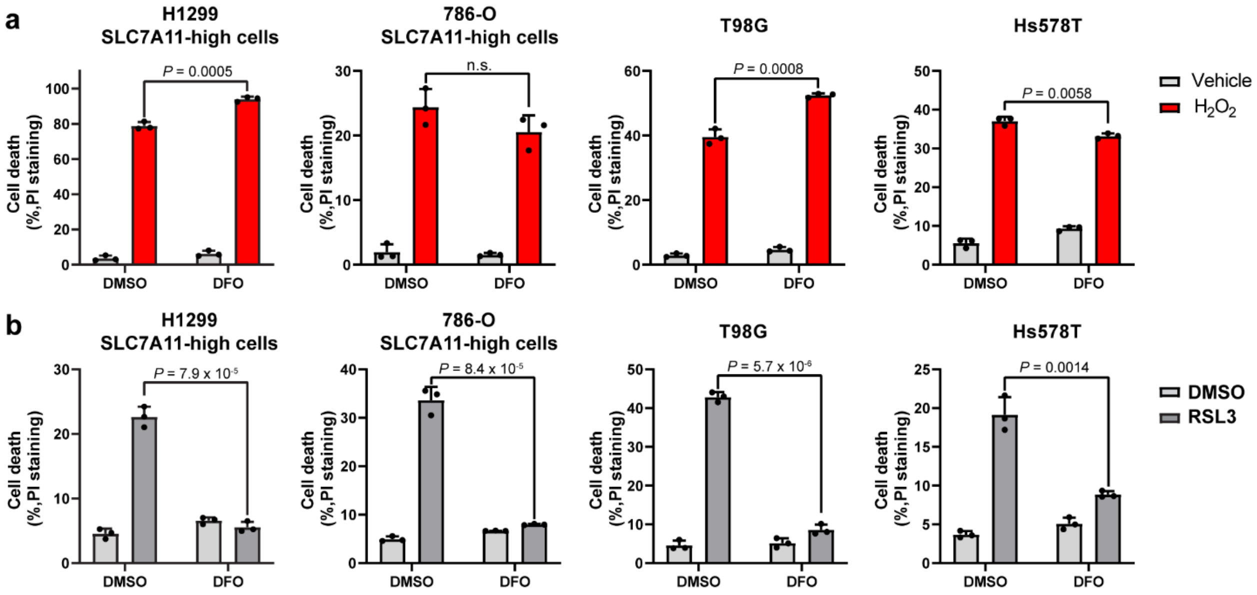

Supplementary Figure 6. Iron chelator treatment does not suppress cell death induced by high SLC7A11 expression under H_2O_2 treatment. a, Cell death in response to treatment with 1 mM H_2O_2 with or without 200 nM deferoxamine (DFO) in SLC7A11-high H1299, SLC7A11-high 786-O, T98G, and Hs578T cells measured using propidium iodide (PI) staining. **b**, Cell death in response to treatment with RSL3 with or without 200 nM DFO in SLC7A11-high H1299, SLC7A11-high 786-O, T98G, and Hs578T cells measured using PI staining. Data were presented as mean ± SD. n indicates independent repeats. P value was determined by two-tailed unpaired Student’s t test. n.s., not significant. Source data are provided as a Source Data file.

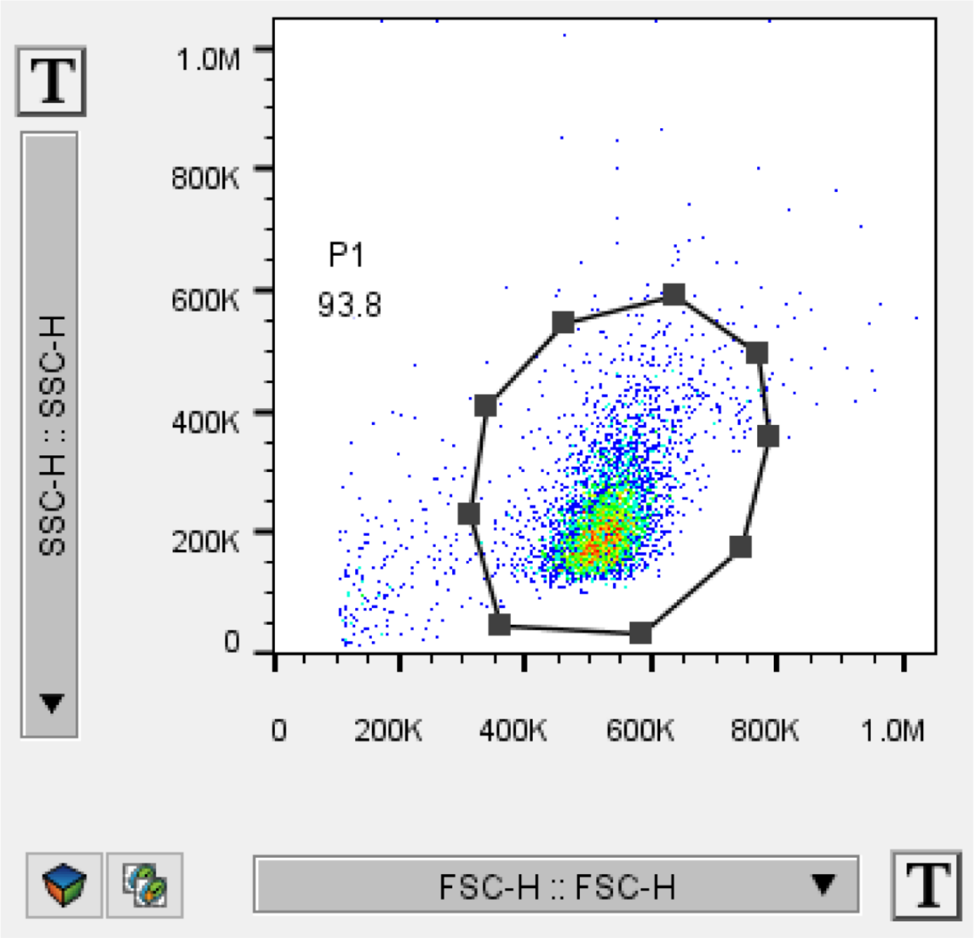

Supplementary Figure 7. Gating strategies for flow cytometry. Initial cell population gating (FSC-H vs. SSC-H) was used to make sure only single cells were used for analysis.

Supplementary Table 1. Oligo sequences for CRISPR-Cas9 knockout.

sgRNA name	sgRNA sequence
sgCtrl	GGCACTACCAGAGCTAACTCA
sg*SLC7A11* #3	GAAGTATTACGCGGTTGCCAC
sg*SLC7A11* #4	GGTGTTCTGGAGCACGCCCTT
sg*BAX* #1	GTGAGCAGATCATGAAGACAG
sg*BAX* #2	GCGAGTGTCTCAAGCGCATCG
sg*BAK* #1	GGCAGGTAGCCCAGGACACAG
sg*BAK* #2	GGGAACTCTGAGTCATAGCGT
sg*GPX1*	GGGGGTCGGTCATAAGCGCG
