# Supplementary material for: SLC7A11 expression level dictates differential responses to oxidative stress in cancer cells
Source: Nat Commun. 2023 Jun 21;14:3673. doi: 10.1038/s41467-023-39401-9 (PMC10281978; doi:10.1038/s41467-023-39401-9)
Supplement: Supplementary file 3 — Reporting Summary [file 41467_2023_39401_MOESM3_ESM.pdf]

nature portfolio

Corresponding author(s): Boyi Gan

Last updated by author(s): May 15, 2023

Reporting Summary

Nature Portfolio wishes to improve the reproducibility of the work that we publish. This form provides structure for consistency and transparency in reporting. For further information on Nature Portfolio policies, see our Editorial Policies and the Editorial Policy Checklist.

Statistics

For all statistical analyses, confirm that the following items are present in the figure legend, table legend, main text, or Methods section.

n/a	Confirmed	
☐	☒	The exact sample size (*n*) for each experimental group/condition, given as a discrete number and unit of measurement
☐	☒	A statement on whether measurements were taken from distinct samples or whether the same sample was measured repeatedly
☐	☒	The statistical test(s) used AND whether they are one- or two-sided<br>*Only common tests should be described solely by name; describe more complex techniques in the Methods section.*
☒	☐	A description of all covariates tested
☒	☐	A description of any assumptions or corrections, such as tests of normality and adjustment for multiple comparisons
☐	☒	A full description of the statistical parameters including central tendency (e.g. means) or other basic estimates (e.g. regression coefficient) AND variation (e.g. standard deviation) or associated estimates of uncertainty (e.g. confidence intervals)
☐	☒	For null hypothesis testing, the test statistic (e.g. *F*, *t*, *r*) with confidence intervals, effect sizes, degrees of freedom and *P* value noted<br>*Give P values as exact values whenever suitable.*
☒	☐	For Bayesian analysis, information on the choice of priors and Markov chain Monte Carlo settings
☒	☐	For hierarchical and complex designs, identification of the appropriate level for tests and full reporting of outcomes
☐	☒	Estimates of effect sizes (e.g. Cohen's *d*, Pearson's *r*), indicating how they were calculated

Our web collection on statistics for biologists contains articles on many of the points above.

Software and code

Policy information about availability of computer code

Data collection	Accuri C6 (BD Bioscience) and Attune NxT Flow Cytometer was used to collect flow cytometry data. The LC-MS complete platform consists of an Accela 1250 HPLC<br>system, Accela Open Autosampler, MayLab Mistraswitch column oven and Exactive orbitrap mass spectrometer, controlled by the Xcalibur 3.0.63 software package was used to collect metabolite data.
Data analysis	GraphPad 9 were used for bar graphs output and statistic analysis. FlowJo_V10 was used for flow cytometry data analysis. Raw HPLC-MS data files (Thermo RAW format) were converted to mzXML files for analysis using msconvert, which is part of the ProteoWizard V3 package. The living image of mice are taken and quantified by IVIS 200 or IVIS Lumina Imaging System. The software and algorithms for data analysis used in this study are all well-established from previous work. All software and<br>custom arguments are included in Methods section. There is no unreported algorithm used in this manuscript.

For manuscripts utilizing custom algorithms or software that are central to the research but not yet described in published literature, software must be made available to editors and reviewers. We strongly encourage code deposition in a community repository (e.g. GitHub). See the Nature Portfolio guidelines for submitting code & software for further information.

Data

Policy information about availability of data

All manuscripts must include a data availability statement. This statement should provide the following information, where applicable:

- Accession codes, unique identifiers, or web links for publicly available datasets
- A description of any restrictions on data availability
- For clinical datasets or third party data, please ensure that the statement adheres to our policy

The uncropped films for immunoblots used in this study are shown in Source Data files.
All data were available within the paper and Source Data file. Source data are provided with this paper.

Human research participants

Policy information about studies involving human research participants and Sex and Gender in Research.

Reporting on sex and gender	N/A
Population characteristics	N/A
Recruitment	N/A
Ethics oversight	N/A

Note that full information on the approval of the study protocol must also be provided in the manuscript.

Field-specific reporting

Please select the one below that is the best fit for your research. If you are not sure, read the appropriate sections before making your selection.

[x] Life sciences [ ] Behavioural & social sciences [ ] Ecological, evolutionary & environmental sciences

For a reference copy of the document with all sections, see nature.com/documents/nr-reporting-summary-flat.pdf

Life sciences study design

All studies must disclose on these points even when the disclosure is negative.

Sample size	The sample size for each experiment is indicated in figures or figure legends. The sample sizes were not predetermined by statistical tools. The sample or group sizes of the experiments were chosen based on previous experience in the lab.
Data exclusions	For xenograft in vivo experiments, mice were excluded from the analysis if mice died prior to the end point of the experiment.
Replication	Multiple independent repeats were included for related experiments. Each experiment was performed for at least twice to make sure similar results are reproducible.
Randomization	For the xenograft studies, 6-8 week female nude mice were chosen as xenograft hosts and randomly allocated into experimental groups. For the metastatic models, 6-7 week NSG mice were chosen as hosts and randomly allocated into experimental groups.No randomization was applied on cell-based experiments because there were defiend groups, e.g, SLC7A11-low vs. SLC7A11-moderate vs. SLC7A11-high.
Blinding	For cell-based experiments, western blotting, Mass spectrometry and FACs, cell types were known when prepare the samples or start to treat cells at the beginning of experiments. Investigators were not blinded during experiments and outcome assessment. There were defined groups and blinding wea not necessary.

Reporting for specific materials, systems and methods

We require information from authors about some types of materials, experimental systems and methods used in many studies. Here, indicate whether each material, system or method listed is relevant to your study. If you are not sure if a list item applies to your research, read the appropriate section before selecting a response.

Materials & experimental systems

n/a	Involved in the study	
☐	☒	Antibodies
☐	☒	Eukaryotic cell lines
☒	☐	Palaeontology and archaeology
☐	☒	Animals and other organisms
☒	☐	Clinical data
☒	☐	Dual use research of concern

Methods

n/a	Involved in the study	
☒	☐	ChIP-seq
☐	☒	Flow cytometry
☒	☐	MRI-based neuroimaging

Antibodies

Antibodies used	Vinculin (1:2000, Sigma-Aldrich, #V4505), SLC7A11 (1:1000, CST, #12691S), SLC7A9 (1:1000, Thermo Fisher, PA5-50887), SLC3A1 (1:1000, Abcam, ab196552), GPX1 (1:1000, CST, 3286S), FLAG (1:1000, Sigma, F1804), BAX (1:1000, CST, #2772T), and BAK (1:1000, CST, #12105T).
Validation	All antibodies used in our study have been validated and detailed information could be found on the website from manufactures as listed below. Some of them have also been validated by our experiments as shown in this manuscript using either overexpress, knockout or knockdown strategies.<br>Vinculin, https://www.sigmaaldrich.com/catalog/product/sigma/v4505?lang=en&region=US<br>SLC7A11, https://www.cellsignal.com/products/primary-antibodies/xct-slc7a11-d2m7a-rabbit-mab/12691<br>BAX, https://www.cellsignal.com/products/primary-antibodies/bax-antibody/2772?site-search-type=Products&N=4294956287&Ntt=bax&fromPage=plp<br>BAK, https://www.cellsignal.com/products/primary-antibodies/bak-d4e4-rabbit-mab/12105?site-search-type=Products&N=4294956287&Ntt=bak&fromPage=plp<br>SLC7A9, https://www.thermofisher.com/antibody/product/SLC7A9-Antibody-Polyclonal/PA5-110394<br>SLC3A1, https://www.abcam.com/products/primary-antibodies/slc3a1-antibody-ab196552.html<br>GPX1, https://www.cellsignal.com/products/primary-antibodies/gpx1-c8c4-rabbit-mab/3286<br>FLAG, https://www.sigmaaldrich.com/US/en/product/sigma/f1804

Eukaryotic cell lines

Policy information about cell lines and Sex and Gender in Research

Cell line source(s)	H1299 (CRL-5803), 786-O (CRL-1932), A498(HTB-44), H226 (CRL-5826), A549(CRL-7909), T98G(CRL-1690), Hs578T(HTB-126) and HEK293T (CRL-3216) cancer cell lines were obtained from ATCC. UMRC6 (#08090513) was purchased from Sigma.
Authentication	Cell line were not authenticated.
Mycoplasma contamination	All cell lines tested negative for mycoplasma contamination.
Commonly misidentified lines (See ICLAC register)	No ICLAC cell line was used in this study.

Animals and other research organisms

Policy information about studies involving animals; ARRIVE guidelines recommended for reporting animal research, and Sex and Gender in Research

Laboratory animals	Athymic nude and NOD scid gamma (NSG) female mice at 6-8-week-old were purchased from ERO mouse facility in MD Anderson Cancer Center or The Jackson Laboratory. Mice were housed under specific-pathogen-free conditions with a 12 h light–12 h dark cycle. The ambient temperature was 21–23 °C, with 45% humidity and the mice had ad libitum access to water and food.
Wild animals	No wild animals involved in this study.
Reporting on sex	No sex were considered in this study.
Field-collected samples	This study didn't involve samples collected from field.
Ethics oversight	All the xenograft model experiments were performed in accordance with a protocol approved by the Institutional Animal Care and Use Committee and Institutional Review Board at The University of Texas MD Anderson Cancer Center. The study is compliant with all relevant ethical regulations regarding animal research.

Note that full information on the approval of the study protocol must also be provided in the manuscript.

Flow Cytometry

Plots

Confirm that:

- [x] The axis labels state the marker and fluorochrome used (e.g. CD4-FITC).
- [x] The axis scales are clearly visible. Include numbers along axes only for bottom left plot of group (a 'group' is an analysis of identical markers).
- [x] All plots are contour plots with outliers or pseudocolor plots.
- [x] A numerical value for number of cells or percentage (with statistics) is provided.

Methodology

Sample preparation	Cells cultured in 12-well plates were incubated with media containing 4 µM ROS dye CM-H2DCFDA (Life Technologies, #C6827) for 30 minutes at 37℃. Then cells were trypsinized and collected into 1.5 ml tubes. After washing once with PBS, cells were resuspended in cold PBS and subjected to flow cytometry analysis. For cell death measurement, cells were stained in PBS with PI for 5 minutes before flow cytometry analysis.
Instrument	Accuri C6 (BD Bioscience) or Attune NxT Flow Cytometer
Software	Using Accuri C6 or Attune NxT Flow software to collect data and FlowJo_V10 software to analyze data.
Cell population abundance	At least 5000 cells were analyzed for each sample.
Gating strategy	Initial cell population gating (FSC-Height Vs SSC-Height) was adopted to make sure doublet exclusion and only single cell was used for analysis. A figure exemplifying the gating strategy would be provided upon request.

- [x] Tick this box to confirm that a figure exemplifying the gating strategy is provided in the Supplementary Information.
